# Pigment production by *Fusarium solani* BRM054066 and determination of antioxidant and anti-inflammatory properties

**DOI:** 10.1186/s13568-020-01054-y

**Published:** 2020-07-01

**Authors:** Bruna S. Menezes, Lucas S. Solidade, Aparecido A. Conceição, Manoel N. Santos Junior, Patrícia L. Leal, Edy S. de Brito, Kirley M. Canuto, Simone Mendonça, Félix G. de Siqueira, Lucas M. Marques

**Affiliations:** 1 Instituto Multidisciplinar em Saúde, Universidade Federal da Bahia. Vitória da Conquista, Bahia, Brazil; 2 Embrapa Agroindústria Tropical, Fortaleza, Ceará Brazil; 3 Embrapa Agroenergia, Distrito Federal, Brasília, Brazil

**Keywords:** Natural red pigment, Submerged fermentation, Doehlert design, Bioactive compounds

## Abstract

The fungal kingdom has been widely studied as a source of bioactive compounds of interest to the pharmaceutical and food industry. This paper studies the production of natural red pigments by *Fusarium solani* BRM054066 in the submerged fermentation system, using Doehlert experimental design to determine optimal cultivation conditions. The chemical composition of the red pigment was determined by Nuclear Magnetic Resonance spectroscopy (NMR) and Ultra-Performance Liquid Chromatography coupled to Mass Spectrometry (UPLC-MS). Antioxidant activity was assessed by the ability to sequester of free radical DPPH. In the analysis of anti-inflammatory activity, murine peritoneal macrophages activated by LPS were used, and the gene expression of TNF-α, IL-1β, IL-6, IL-10 and IL-17 was determined using qPCR. As a result, it was found that agitation at 200 rpm and glucose concentration ≥ 20 g/L promote the best results in the production of red pigment. The chemical compounds identified were two naphthoquinones, fusarubin and dihydrofusarubin, and an anthraquinone, a bostrycoidin, being fusarubin the majority compound. The red pigment showed antioxidant activity by scavenge 50% of the DPPH radical, in a concentration of 24 µg/mL. The pigment also showed an effective anti-inflammatory capacity by reducing the overexpression of the pro-inflammatory cytokines TNF-α, IL-1β and IL-6 and promoting the production of anti-inflammatory IL-10 and IL-17, in murine macrophages activated by LPS (p < 0.05). According to the results, the fungus *F. solani* BRM054066, under optimized conditions of cultivation, proved to be a promising source of biologically active natural pigments with wide industrial applicability.

## Key points

- This study optimized the production of natural pigment using biotechnological tools
- Pigment has promising properties against oxidative stress and inflammatory disorders
- Pigment has the potential to be applied as food additive and in cosmetic industry

## Introduction

The search for natural pigments as an alternative to potentially harmful synthetic dyes, especially for applications in the food, cosmetic and textile industries has dramatically increased (Sivakumar et al. 2009). One of the main advantages of natural dyes is their low environmental impact during production. Furthermore, natural pigments not only improve the marketability of a product but also add extra features such as antimicrobial, and antioxidant properties (Tuli et al. 2015). Antioxidants are molecules that delay or inhibit cell damage by donating electrons to a disorderly free radical and neutralizing them through their free radical scavenging properties (Lobo et al. 2010). Thus, antioxidant agents can act to protect food, in addition to providing extra benefits to consumers, since the increase of free radicals in the body increases the chances of the occurrence of chronic diseases, such as cancer, diabetes, cardiovascular diseases and autoimmune (Rankovic et al. 2011). So, the combination of antioxidant and anti-inflammatory effects of a natural pigment used as a food additive would be very promising, since it would give it protective properties of cellular constituents against oxidative stress and against tissue damage caused by inflammatory disorders, and it can be effective in preventing numerous diseases. However, the major challenge of replacing synthetic dyes with natural ones, especially those extracted from plants such as tannins, flavonoids, and quinonoids, is the high cost of production (Kasiri and Safapour 2013). In this regard, a submerged culture of microorganisms appears as an efficient biotechnological tool for the production of natural pigments. By means of this methodology it is possible to obtain high yields of production, in a short time and reduced space requirement, optimizing its production for industrial applicability (Manan et al. 2017).

Naphthoquinones are pigments derived from naphthalene, belonging to the quinone family, which exhibit a variety of chemical structures and demonstrate various biological activities. These substances are secondary metabolites, found in higher plants, bacteria, and fungi (Soares et al. 2017; Stodůlková et al. 2015). The *Fusarium* genus has been reported as a producer of a naphthoquinone known as fusarubin, with *Fusarium solani* being the most studied species (Ammar et al. 1979; Shah et al. 2017). *F.solani* is a large species complex that includes some important pathogenic fungi, which are associated with human and animal diseases, as well as a variety of saprophytic and endophytic species (Summerell et al. 2011). Several studies have proven its ability to produce fusarubin and derivatives (*O*-ethylfusarubin, hydroxydihydrofusarubin and *O*-ethylhydroxydihydrofusarubin (Gerber and Ammar 1979), as well as anthraquinones such as bostrycoidin (Dame et al. 2016).

Fusarubin and bostrycoidin are considered to be biosynthesized by the formation of a C14 heptaketide aldehyde as a common intermediate. Polyketides are produced by multi-domain polyketide synthase (PKS). This linear intermediate undergoes a cyclization catalyzed by the NhPKS1 enzyme, indicating the involvement of the enzyme in the biosynthesis of these molecules (Studt et al. 2012; Awakawa et al. 2012). Fusarubin and bostrycoidin, and many of the intermediates of the biosynthetic pathway, have demonstrated a wide range of biological activities, including antimicrobial and antitubercular activities (Shah et al. 2017), antifungal (Frandsen et al. 2016) and anticancer (Chowdhury et al. 2017), and potential cytotoxicity against human leukemia cells (K-562), besides presenting prominent activity against *Staphylococcus aureus, Escherichia coli, Pseudomonas aeruginosa* and *Bacillus megaterium* (Khan et al. 2016).

Due to the presence of biologically active substances in pigments produced by *Fusarium* species, the present work aimed at optimizing pigment production of the fungus *Fusarium solani* BRM054066 from culture in submerged fermentation. The Doehlert experimental design was used to determine the optimal conditions of agitation and initial glucose concentration in function of culture time for pigment production. NMR and UPLC-MS were utilized to determine the chemical composition of the produced red pigment. Antioxidant and anti-inflammatory properties was investigated to assess the pigment’s potential for possible applications in the food, pharmaceutical or cosmetic industries.

## Materials and methods

### Microorganism

The *Fusarium solani* BRM054066 strain was obtained from the collection of Embrapa Agroenergia [*Coleção de Microrganismo e Microalgas Aplicadas a Agroenergia e Biorrefinaria da Embrapa Agroenergia* (CMMAABio). Access: http://alelomicro.cenargen.embrapa.br/InterMicro/Passaporte/buscaBRM.xjs?de=54066&ate=&Buscar=Buscar#], from Brasília, Distrito Federal, Brasil. The mycelium was kept under refrigeration (7 °C) in Petri dishes containing potato dextrose agar (PDA) medium.

### Experimental design

The optimization process of pigment production by *F. solani* BRM054066 was carried out by response surface methodology using the Doehlert experimental design. The independent variables applied were agitation, initial glucose concentration and incubation time. Doehlert array was selected due to its uniform distribution of experiments within a chosen experimental region. Moreover, Doehlert designs are rotatable (constant variance at equal distance from the central point), offering the possibility of investigating adjacent experimental domains and adding new variables in a stepwise manner, and the number of required experiments is smaller than that for other designs (such as central composite and Box–Behnken designs), while achieving a similar precision of the estimates of the coefficients of the quadratic model. A Doehlert matrix can be represented in normalized independent variables by the vertices and center of a hexagon (Pinkowska 2014). A Doehlert matrix can be associated with a quadratic polynomial model that takes the following form:

$$ \varvec{y } = \varvec{ \beta }0\varvec{ } + \varvec{ \beta i}.\varvec{Xi } + \varvec{ \beta j}.\varvec{Xj } + \varvec{ \beta ii}.\varvec{Xi}2\varvec{ } + \varvec{ \beta jj}.\varvec{Xj}2\varvec{ } + \varvec{ \beta ij}.\varvec{Xi}.\varvec{Xj} $$

where:

- **y–**Represents the expected response, i.e., the pigment concentration;
- **β**_**0**_**, β**_**i**_**, β**_**j**_**, β**_**ii**_**, β**_**jj**_**, β**_**ij**_–are coefficients of the model;
- **X**_**i**_ and **X**_**j**_-are independent variables.

The standard deviations of the experimental responses were evaluated by repeating the experiments in the centers of the experimental domains three times. STATISTICA (version 10, Stat Soft) was employed for regression analysis and for estimating the regression coefficients. The statistical significance of the model was determined by the Fisher’s test through ANOVA (analysis of variance). Calculations were done at a 95% confidence level.

### Effect of agitation and fermentation time on pigment production

Disks of PDB (15 mm) containing *F. solani* BRM054066 mycelium was inoculated on 250 ml Erlenmeyer flasks containing 100 ml of POL culture medium [5 g/L (NH_4_)_2_SO_4_, 0.2 g/L MgSO_4_·7H_2_O, 1.0 g/L K_2_HPO_4_, 2.0 g/L yeast extract, 1.0 g/L peptone, 20 g/L glucose] previously sterilized at 121 °C for 15 min and pH adjusted between 6.0 and 6.5. The flasks were incubated in an orbital shaker at 25 °C. The effect of incubation time was observed at different growth days 1, 4, 7, 10, and 13 and different agitations 0 (static), 100 and 200 rpm). The real values and corresponding coding values are found in Additional file 1: Table S1. At the end of cultivation, the samples were centrifuged at 5000 rpm for 10 min obtaining supernatant and fungal biomass. The supernatants were stored in a freezer at − 60 °C for further pigment extraction.

### Effect of initial glucose concentration and cultivation time on pigment production

To evaluate the influence of initial glucose concentration on pigment production by *F. solani* BRM054066, different glucose amounts were used for POL medium preparation. About 2, 7, 12, 17 and 22 g/L were added to the POL medium and incubation time was analyzed at 3, 6 and 9 days, as described in Additional file 1: Table S2. The cultivation process followed the same protocol reported in the previous item on the Effect of initial glucose concentration and cultivation time on pigment production, applying the variations of the glucose concentration in the POL medium and fermentation time as described in the Additional file 1: Table S2. The best agitation observed in 2.2.1 section was maintained for glucose effects on pigment production.

### Pigment extraction and quantification

The extraction of pigment was done with chloroform (v/v). For this, a 3 mL of the supernatant culture was mixed with the same volume of chloroform in test tubes. After phase separation, the aqueous portion was discarded, and the chloroform samples containing the pigment were transferred to unopened vials and preserved at room temperature for 48 h, for complete evaporation of the solvent. The dry pigment was weighed, and the pigment concentration in the samples was calculated.

### Identification of pigment components

Ultra-Performance Liquid Chromatography coupled to Mass Spectrometry analyses (UPLC-MS) were carried out on a UPLC-QTOF Acquity Xevo system (Waters) fitted with an electrospray ionization interface (ESI). Separations were performed on a Waters Acquity BEH C18 column (150 mm × 2.1 mm, 1.7 μm). The mobile phase was composed of H_2_O (A) and acetonitrile (B), each containing formic acid (0.1% v/v). The elution gradient ranged from 2% to 95%, at a flow rate of 500 μL min^−1^. The fractions were analyzed in the positive (PI) and negative (NI) ionization modes in a range of 100–1200 Da. ESI conditions were defined as follows: 2800 V capillary voltage, 40 V cone voltage, source temperature at 120 °C, desolvation temperature at 330 °C, 20 L h^−1^ gas flow, 600 L h^−1^ desolvation gas flow, and MCP (microchannel plate voltage) detector at 1900 V. The identification of the compounds was done through the following parameters: (1) molecular formula deduced from the exact mass (4 decimal places), considering a mass error of 5 ppm; (2) the isotopic ion pattern (i-fit) and (3) the ion fragmentation pattern compared to literature data.

Hydrogen-1 and Carbon-13 Nuclear Magnetic Resonance (^1^H and ^13^C NMR) analyses, including one (1D) and two-dimensional (2D) experiments, were accomplished on an Agilent DD2 spectrometer (14.1 T), equipped with a 5 mm inverse detection probe, operating at frequencies of 599.56 and 150.77 MHz for the ^1^H and ^13^C, respectively. Samples were dissolved in 600 μL of deuterated chloroform (CDCl_3_, Cambridge Isotope Laboratories) and analyzed in 5 mm glass tubes. The chemical shifts (δ) were expressed in parts per million (ppm) and referenced to the hydrogen signal of the non-deuterated residual molecules of the deuterated solvent (δH 7.26) and by the central carbon peak of the deuterated solvent (δC 77.23). The experiments were performed at 26 °C. In the ^1^H and ^13^C 1D NMR experiments, the following values were established for the acquisition parameters, respectively: spectral widths of 16 and 252 ppm, acquisition time of 1.7 s and 0.865 s, 45o pulse widths of 4.15 μs and 3.20 μs (58 dB), number of 16 and 32 K transients, and relaxation time of 1 s. The 2D spectra of homonuclear correlation (^1^H,^1^H COSY-Correlations Sectroscopy) and heteronuclear (^1^H,^13^C HSQC-Heteronuclear Single Quantum Coherence and HMBC-Heteronuclear Multiple Bond Correlation) were acquired with field gradient and standard pulse sequence, employing a number of transients of 16 and 32, respectively. In COSY, 897 × 128 points were used for the acquisition data matrix and 4096 × 4096 points for processing, whereas for HSQC and HMBC experiments, 1142 × 256 points were used in the acquisition and 4096 × 2048 points in the processing.

### Antioxidant activity by DPPH method

The antioxidant activity of the pigment was determined by the ability to sequester 1,1-diphenyl-2-picrylhydrazyl (DPPH) radical, according to the method described by Brand-Williams et al. (1995), with modifications according to Kim et al. (2002). A volume 100 μL aliquots of different pigment dilutions and 3.9 mL of a methanolic solution of DPPH were added in different test tubes. The tubes were homogenized and allowed to stand in the dark for 20 min. At the end, absorbance was measured at 517 nm. The DPPH scavenging activity was calculated as followed.

$$ \varvec{DPPH scavenging } = \varvec{ }\left[ {\left( {\varvec{A}0\varvec{ } - \varvec{ A}1} \right)/\varvec{A}1} \right]\varvec{ \times }100 $$

where:

- **A**_**0**_ = absorbance of control (pure DPPH),
- **A**_**1**_= absorbance of the samples.

The concentration of pigment required to reduce 50% of the initial DPPH radicals (EC_50_) was calculated from linear regression analysis. The standard curve was constructed using solutions of ascorbic acid in the same concentrations of the pigment. All analyses were performed in triplicate.

### Anti-inflammatory activity

Macrophages were obtained from the peritoneal cavity of mice stimulated intraperitoneally with 3% thioglycollate. After 3 days of stimulation, the animal was sacrificed and the peritoneal lavage was obtained by inoculating 5 ml of complete RPMI medium. Cells were cultured in RPMI supplemented with 10% fetal bovine serum containing 50 μg/mL ciprofloxacin. The macrophages were incubated for 24 h in a CO_2_ oven at 37 °C, in 24-well cell culture plates, with a cell density of 10^6^ per well. Subsequently, the cells were treated with different pigment concentrations (30, 20, 10 and 5 μg/mL), 1 h prior to the addition of the LPS stimulant. As a positive control, cells were treated with only LPS, and for the negative control, cells were treated with only phosphate buffered saline (PBS). The experiment was carried out in triplicate. The culture plate was maintained in a CO_2_ incubator, at 37 °C for 6 h. After the period, culture media and macrophages were collected in *Eppendorf*-*like* tubes, separately, and stored at − 80 °C for further analysis of gene expression. A customized qPCR Primer assay was performed to evaluate the gene expression of the cytokines IL-1β, IL-6, IL-10, IL-17 and TNF-α. The reaction was performed in a StepOnePlus Real-Time PCR System (Applied Biosystems, Brazil) with SYBR Green (Qiagen-SA Bioscience, Brazil), using the program recommended by the manufacturer. Analysis of gene expression data was performed using the 2^−ΔΔ*CT*^ method (Rao et al. 2013). GAPDH was used as an endogenous gene to evaluate the overall cDNA content.

### Statistical analyses

The results were expressed as mean values ± standard deviation. Multiple comparisons were performed using GraphPad Prism 7 software (GraphPad Software, San Diego, CA-USA) using the Anova parametric test, followed by Bonferroni post-test. Differences were considered significant at *p* < 0.05.

## Results

### Influence of agitation, glucose concentration, and cultivation time on pigments production by *F. solani* BRM054066

The first experimental design is presented in Table 1. The production of pigment ranged from 0.00 to 276.00 μg/mL. Table 2 shows the estimated effects of each variable, as well as the interaction, in pigment concentration. It can be observed that all effects, except the interaction between them, presented statistical significance at 95% confidence level, with the linear effect of the agitation being the most expressive.

**Table 1:** Doehlert two-factor experimental design (agitation and time) along with experimental and predicted values

Experiment number	*x*_*A*_	*x*_*T*_	*X*_*A*_	*X*_*T*_	Pigment concentration μg/mL	
					Experimental	Predicted
1	0.866	− 0.5	200	4	192.22	203.16
2	0.866	0.5	200	10	276.00	265.06
3	0	− 1	100	1	0.00	− 15.01
4	0	0	100	7	140.67	147.18
5	0	0	100	7	147.52	147.18
6	0	0	100	7	153.34	147.18
7	0	1	100	13	93.78	108.79
8	− 0.866	− 0.5	0	4	34.06	53.14
9	− 0.866	0.5	0	10	134.12	115.04

**xA**: Coded Agitation variable; **xT:** Coded Time variable; **XA:** Agitation (RPM). **XT**: Time (days)

**Table 2:** Estimated effect of independent variables (agitation and incubation time) on pigment production

Factor	Effect	Standard error	*t*(2)	p
Mean	147.1767	3.661540	*40.1953*	*0.000618*^*a*^
*X*_*A*_	150.0200	6.341974	*23.6551*	*0.001782*^*a*^
*X*_*A*_^*2*^	73.9900	8.684105	*8.5202*	*0.013497*^*a*^
*X*_*T*_	61.9000	3.661540	*16.9055*	*0.003481*^*a*^
*X*_*T*_^*2*^	**− **50.1433	2.894702	*− 17.3225*	*0.003316*^*a*^
*X*_*A* x_*X*_*T*_	**− **8.1400	6.341974	*− 1.2835*	*0.327940*

***X***_***A***_: Agitation variable; ***X***_***T***_: Time variable. ***t***: t-Value in Student’s *t* test. **p**: *p*-Value

^a^Significant factors at 95% of confidence level

The regression model for the effects of agitation and cultivation time, ignoring its interaction, on pigment production is presented in following equation.

1 $$ \varvec{ }\left[ {\varvec{Y } = \varvec{ }147.177 + \varvec{ }86.617\varvec{XA } + \varvec{ }61.900\varvec{XT } + \varvec{ }49.330\varvec{XA}2\varvec{ }{-}\varvec{ }100.287\varvec{XT}2} \right] $$

where:

- **Y** is the expected response, the pigment concentration;
- **X**_**A**_ and **X**_**T**_ are the independent variables, agitation and time respectively.

**Fig. 1:**
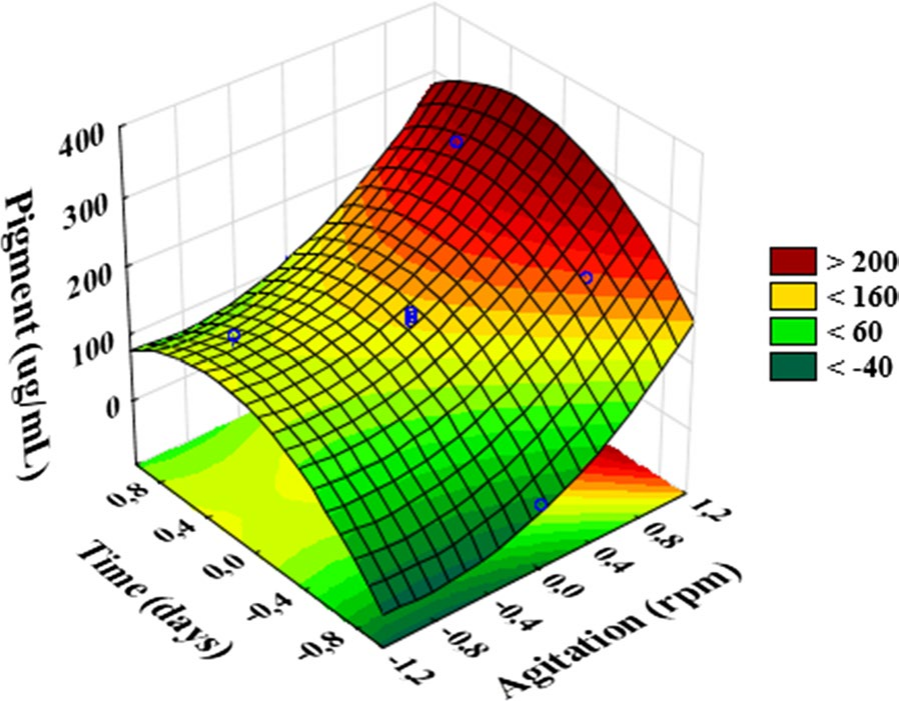
Surface response graphs for pigment concentration as a function of agitation and time cultivation

According to ANOVA analysis (Table 3), the regression model was significant at the confidence level considered (95%), as a satisfactory correlation coefficient (R^2^ = 0.97) was obtained, and the *F*-*value* of the regression was greater than the listed F-value. In addition, the *F*-*value* of the lack of fit was lower than the listed *F*-*value*, showing that the model is reliable. The surface response graph, obtained using the fitted model presented in Eq. 1, is presented in Fig. 1. According to the surface graph presented the pigment production was maximized when higher agitation rates were employed.

**Table 3:** Analysis of variance (ANOVA) of the regression mode’l (Eq. 1)

Source of variation	Sum of squares	Degree of freedom	Mean squares	F-value	F-tab^a^
Regression	52087.40	4	13021.85	34.76	6.39
Residual	1498.50	4	374.6254		
Lack of fit	1418.06	2	709.0301	17.63	19.00
Pure error	80.44	2	40.22063		
Total	53585.90	8			

R^2^ = 0.97204; Adjusted R^2^ = 0.94407

^a^Listed F-*value* (95%)

To optimize the pigment production process, a second experimental plan was carried out with initial glucose concentration on culture medium ranging from 2 to 22 g/L, keeping the agitation at 200 rpm. The experimental design and the results are presented in Table 4. The production of pigment by *F. solani* ranged from 3.12 to 150.78 μg/mL.

**Table 4:** factor experimental design (glucose and incubation time) along with experimental and predicted values

Experiment number	*x*_*G*_	*x*_*T*_	*X*_*G*_	*X*_*T*_	Pigment concentration (μg/mL)	
					Experimental	Predicted
1	− 0.5	0.866	7	9	9.34	15.77
2	0.5	0.866	17	9	113.12	106.69
3	− 1	0	2	6	3.12	− 3.31
4	0	0	12	6	38.17	30.63
5	0	0	12	6	23.39	30.63
6	0	0	12	6	30.33	30.63
7	1	0	22	6	150.78	157.21
8	− 0.5	− 0.866	7	3	14.61	21.04
9	0.5	− 0.866	17	3	97.06	90.63

**xG**: Coded Glucose variable; **xT:** Coded Time variable; **XG:** Glucose (g/L); **XT**: Time (days)

Table 5 presents the estimated effects of each variable, as well as its interaction with the pigment concentration, showing that just the effect of glucose concentration was significant at 95% of confidence level, with their linear effect being the most expressive.

**Table 5:** Estimated effect of independent variables (glucose and incubation time) on pigment production

Factor	Effect	Standard error	*t*(2)	p
Mean	30.63000	4.26925	7.17455	0.018879^a^
*X*_*G*_	80.25833	4.26925	18.79915	0.002818^a^
*X*_*G*_^*2*^	23.16000	3.37514	6.86193	0.020584^a^
*X*_*T*_	5.39500	7.39457	0.72959	0.541519
*X*_*T*_^*2*^	32.64500	10.12543	3.22406	0.084228
*X*_*G*_x *X*_*T*_	10.66500	7.39457	1.44228	0.285981

***X***_***G***_: Glucose variable; ***X***_***T***_: Time variable; ***t***: t-Value in Student’s t-test. **p**: *p*-Value

^a^Significant factors at 95% of confidence level

The regression model for the effects of glucose concentration and cultivation time on pigment production is presented in the following equation (Eq. 2), considering only the significant coefficients:

2 $$ \varvec{ Y } = \varvec{ }39.335\varvec{ } + \varvec{ }80.258\varvec{XG } + \varvec{ }41.967\varvec{XG}2 $$

where:

- **Y** is the pigment concentration;
- **X**_**G**_ is the glucose concentration.

**Fig. 2:**
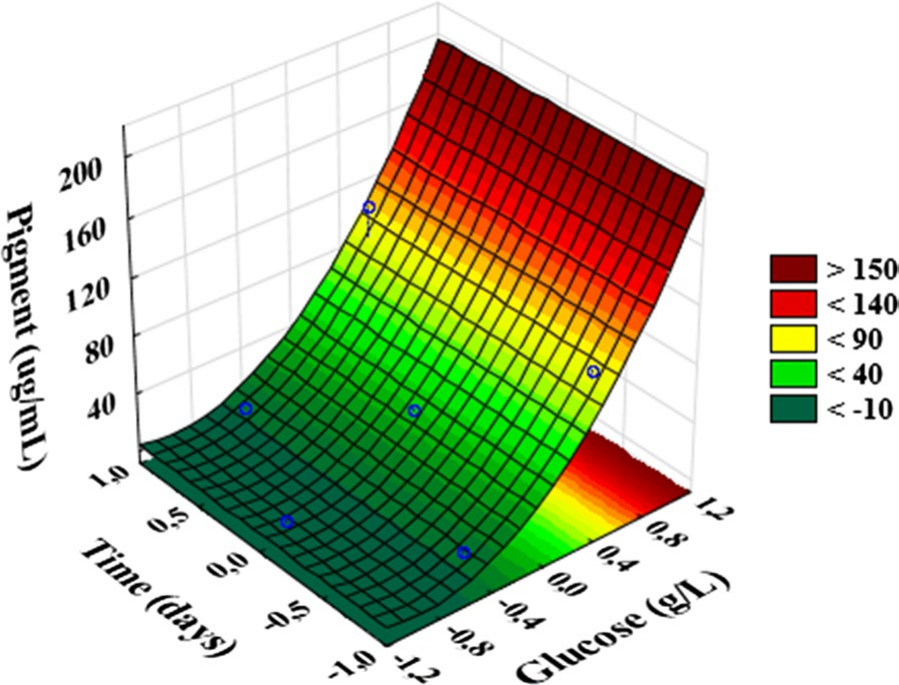
Surface response chart for pigment concentration as a function of glucose concentration and time cultivation

Table 6 report the ANOVA results of the adjusted model, showing that the regression model was significant at the confidence level of 95%, obtaining a satisfactory correlation coefficient (R^2^ = 0.95), the *F*-*value* of the regression greater than the listed *F*-*value* and the *F*-*value* of the lack of fit smaller than the listed *F*-*value*, demonstrating that this model is reliable. Figure 2 presents the surface response graph obtained using the fitted model presented in Eq. 2.

**Table 6:** Analysis of variance (ANOVA) of the regression model (Eq. 2)

Source of variation	Sum of squares	Degree of freedom	Mean squares	F-value	F-tab^*a*^
Regression	21525.77	2	10762.89	60.44	5.14
Residual	1068.52	6	178.0866		
Lack of fit	959.16	4	239.7901	4.39	19.25
Pure error	109.36	2	54.6796		
Total	22594.29	8	2824.286		

R^2^ = 0.95271; Adjusted R^2^ = 0.93694

^a^Listed F-*value* (95%)

### Identification of the pigment

A UPLC-ESI-QTOF-MS method was established to characterize the constituents of *F. solani* BRM 054066 pigment. Compound 1 exhibited a precursor ion [M-H]^−^ at *m/z* 305.0625 corresponding to the molecular formula C_15_H_13_O_7_, then it was tentatively identified as naphthoquinone fusarubin. Furthermore, its MS/MS fragments at *m/z* 305, 29, 248 and 233 matched with those described in literature (Nielsen et al. 2011). Compound 2 gave an ion [M + H]^+^ at *m/z* 286.0715, whose molecular formula was consistent with C_15_H_12_NO_5_, therefore it was annotated as the aza-anthraquinone bostrycoidin. In addition, its MS/MS fragments at *m/z* 286 and 243 matched with those described in literature (Nielsen et al. 2011). The nuclear magnetic resonance (NMR) analysis showed a complex chemical profile, and the chemical shift values were arranged according to the information extracted from the one- and two-dimensional spectra, leading to the characterization of fusarubrin and bostrycoidin.

In the ^1^H-NMR spectrum (600 MHz, CDCl_3_), fusarubin signals were observed at δ 6.18 (1H, s), δ 4.89 (2H, s), δ 3.94 (3H, s), which were related to olefinic, oxymethylenic and methoxyl hydrogens, respectively. The doublets at δ 3.04 and 2.71 (1H, d) and the singlet at δ 1.65 (3H, s) were associated with alicyclic hydrogen and methyl, respectively. In addition, signals at δ 12.93 and 12.65 (3H, s) were characteristic of chelated hydroxyls with carbonyl. In the ^13^C NMR spectrum (150 MHz, CDCl_3_), the fifteen fusarubin resonances were identified, two of which were attributed to ketone carbonyls (δ 184.9 and 178.5), eight associated with aromatic carbons and olefinic (δ 160.9, 160.5, 157.5, 137.3, 133.0, 110.0, 109.9 and 107.8), along with five signals related to alkyl carbons (δ 94.5; 58.8, 56.9, 32.4 and 29.6), characterizing the naphthoquinone skeleton.

The ^1^H,^13^C-HMBC NMR spectrum showed ^1^H, ^13^C correlations at two and three bonds allowing unequivocal identification of the naphtaquinone skeleton and the position of its substituents through the following couplings: olefinic hydrogen at δ 6.18 with the carbonyls at δ 184.9 and 178.5 as well as with the olefinic carbon at δ 107.8; hydroxyls at δ 12.93 and 12.65 with the aromatic carbons at δ 157.5 and 160.5; methoxyl δ 3.94 with the olefinic carbon at δ 160.9; oxymethylenic hydrogens at δ4.89 with the aromatic carbon at δ 137.0; oxymethylene and methylene hydrogens at δ 4.89 and 3.04 with the aromatic carbon signal at δ 137.0, besides methyl and methylene hydrogen signals at δ 1.65 and 3.04 with the hemiketal carbon signal at δ 94.5. In addition, the NMR data were compiled and are in accordance with those from literature (Additional file 1: Table S3) (Kumar et al. 2017; Kurobane et al. 1985).

^1^H NMR spectrum of bostrycoidin showed resonances at δ 13.49; 13.20; 9.50; 7.96; 6.76, 4.02 and 2.80. Due to the low concentration of bostrycoidin in the *F. solani* BRM054066 pigment, only the most intense signals referring to methoxyl (δ 56.8) and methyl (δ 25.3) could be observed yet indirectly through correlations in the HSQC spectrum. The ^1^H NMR data were in agreement with those from literature (Table S3) (Yamamoto et al. 2002).

After identification of fusarubin, we could characterize the presence of another naphthoquinone: dihydrofusarubin. In the UPLC-ESI-qTOF chromatogram, dihydrofusarubin was detected as a precursor ion [M-H]^−^ at *m/z* 307.0820 (C_15_H_13_O_7_). In the NMR spectrum, its signals were less intense than those of fusarubin; however, they could be unambiguously assigned through 2D NMR spectra. Additionally, its chemical shifts were similar to those reported in the literature (Additional file 1: Table S3) (Tatum and Baker 1983). All spectra are available at the supplementary material (Additional file 1: Figure S1–S7).

### Antioxidant activity

DPPH-radicals scavenging activity was used to evaluate the antioxidant capacity of the pigment from *F. solani* BRM054066. The results showed dose-dependent DPPH-scavenging activity (22–375 µg/mL), comparing to the standard curve of ascorbic acid. For estimating the antioxidant activity, the IC_50_ was calculated by linear regression (Additional file 1: Figure S8). The IC_50_ value of pigment was 24 µg/mL.

### Anti-inflammatory activity

**Fig. 3:**
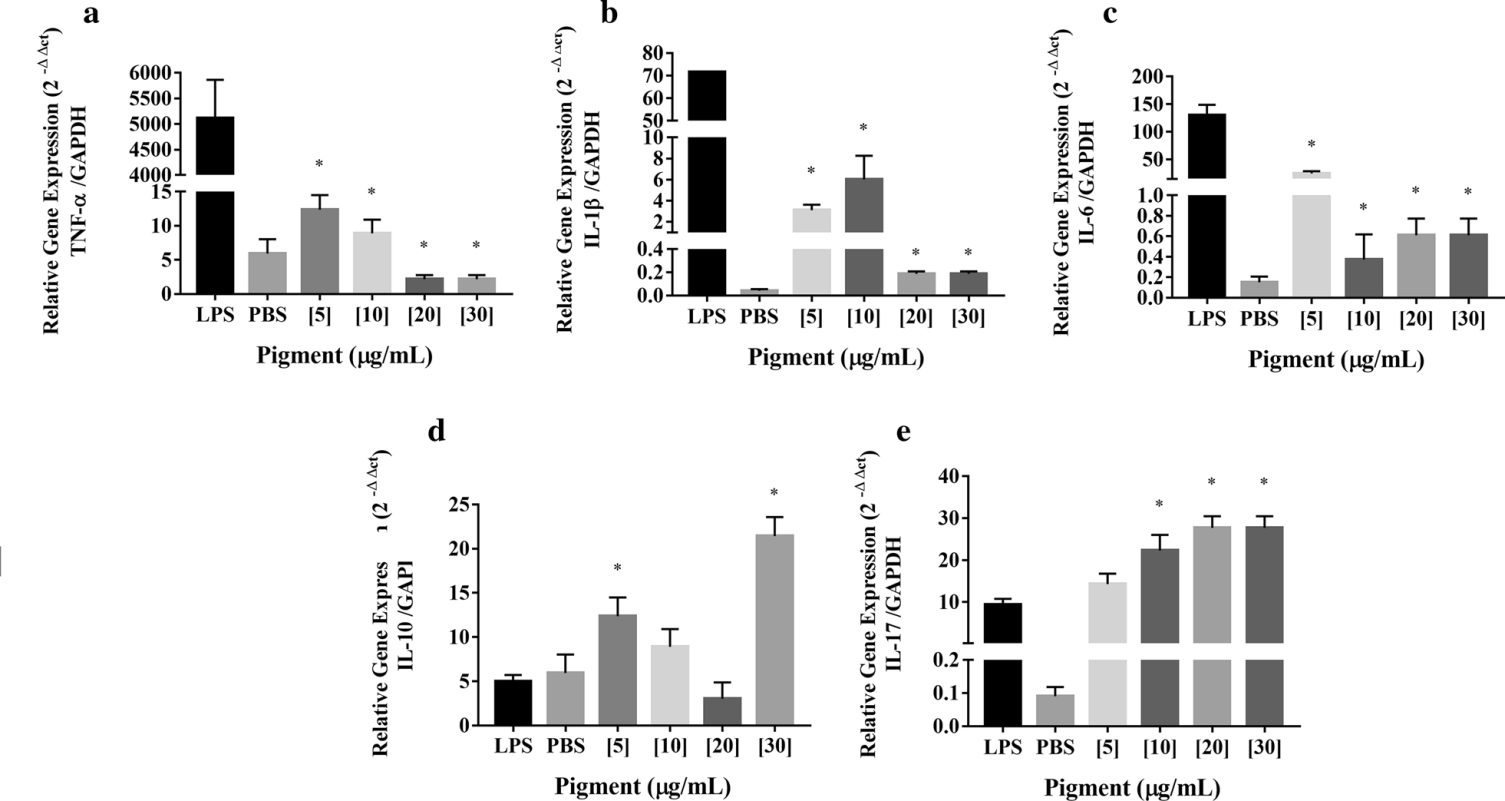
Relative gene expression in murine macrophages stimulated with LPS and treated with the pigment of *F. solani* BRM054066, in different concentrations. **a** Expression of TNF-α. **b** Expression of IL-1β. **c** Expression of IL-6. **d** Expression of IL-10. **e** Expression of IL-17. The treatments were analyzed by the Anova-one way parametric test and Bonferroni post-test (GraphPad Prism^®^ software version 7.0). LPS was used as a positive control and PBS was used as the negative control. Statistical significance was set at *p* < 0.05, represented by the * symbol, compared to the positive control (LPS)

The anti-inflammatory potential of the pigment produced by the *F. solani* BRM054066 in murine macrophages activated by LPS was evaluated. Under basal conditions, macrophages produce low levels of TNF-α, IL-1β, IL-6, whereas stimulation with LPS induces a high production of these pro-inflammatory cytokines. Cytokine expression was altered in the treated groups (Fig. 3). Expressions of TNF-α, IL-1β and IL-6 were drastically reduced in LPS-stimulated macrophages, previously treated with all of the tested pigment concentrations, when compared to the positive control (LPS) (*p* < 0.05). For the anti-inflammatory cytokine IL-10, the concentrations that stimulated its production were 5 and 30 μg/mL (*p* < 0.05). This can be explained by the presence of more than one component in the *F. solani* BRM054066 pigment, where in the optimum concentration for the IL-10 stimulatory effect may be 5 μg/mL for one compound and 30 μg/mL for another. For IL-17, all concentrations from of 10 μg/mL were effective in inducing their expression (*p* < 0.05).

## Discussion

Pigments from natural sources are interesting substitutes for artificial synthetic colorants used in foodstuff, cosmetic and pharmaceutical manufacturing processes (Kim et al. 1995; Cho et al. 2002). There are a number of microorganisms that have the ability to produce pigments in high yields, including such species as *Monascus, Paecilomyces, Serratia, Cordyceps, Streptomyces* and yellow–red and blue compounds produced by *Penicillium herquei* and *Penicillium atrovenetum* (Gunasekaran and Poorniammal 2008). *Fusarium ssp.* are microorganisms that cause plant diseases and some species have complex pigmentation, such as *F. graminearum*, which has several pigments of similar colors, ranging from yellow and orange to red. Some of these compounds have already been identified, such as aurofusarin of the naphtoquinone class, which has a golden yellow color, carotenoids with yellow, orange and red colors, rubrofusarin, of red color, which belongs to the class of naphthopyrones and resembles the aurofusarin (Cambaza 2018), in addition to ″5-deoxybostrycoidin-based melanin″, of a nature related to fusarubins (Frandsen et al. 2016). Evidence indicates that some of these pigments are important for the protection of UV rays and free radicals (Dadachova et al. 2008), and other studies suggest that pigments may be related to phytotoxic activities. Duarte and Archer (2003) showed that *F. solani* isolates that produce red pigments in the culture medium are more efficient in producing secondary metabolites with toxigenic properties, capable of inducing vein discoloration in detached leaves and wilting in transpiring microcuttings in black pepper (*Piper nigrum*), when compared to those that produced pink or white filtrates. In this study, the pigment production by *Fusarium solani* BRM054066 was investigated under submerged fermentation under conditions of optimization of the agitation and initial glucose concentration as a function of the culture time.

The productivity of pigments by microorganisms in submerged cultivation is influenced by the composition of the culture medium and the physical conditions in which they are submitted. Carbon sources provided in the medium play an important role both in growth and in the production of primary and secondary metabolites (Singh et al. 2016). Glucose has been reported as the best substrate for fungal pigment production, especially *Monascus* species (Mostafa and Abbady 2014). In addition, the concentration of glucose in the culture medium directly affects the amount of pigment produced. When the glucose concentration is very high, it inhibits the formation of secondary metabolites, impeding pigment production. It is recommended that glucose be kept below 20 g/L in the culture medium (Manan et al. 2017). Mukherjee and Singh (2011) used variations of glucose concentrations in the culture medium between 10 and 40 g/L for the production of red pigment by *Monascus purpureus*. It was found in the study that glucose at 18 g/L optimized pigment production. Reduced production was observed at glucose levels above this concentration, perhaps due to the predominance of the fermentative metabolism, where increased ethanol production occurs and the respiratory chain enzymes are inhibited (Crabtree Effect). Despite this, in the present work, the best results of the pigment production by *F. solani* BRM054066 were found in glucose concentrations above 20 g/L.

In addition to the composition of the culture medium, pigment production is strongly influenced by the oxygen supply (Yang et al. 2015). The main objective of an agitation system is to promote the transfer of oxygen from the gas phase to the culture medium and consequently to the microorganism, in addition to preventing the sedimentation of the biomass and maintaining suspended solids (Cinbiz et al. 2010). The stirring rate at 200 rpm used in the present work increased the pigment yield of *F. solani* BRM054066 compared to stirring at 100 rpm and without stirring. The production of pigments in a cultivation at high stirring rates was reported. Vendruscolo et al. (2016) investigated the effects of shaking frequency on monascorubrin and rubropunctatin pigment production by *M. Ruber* in a 6 L bioreactor. It was observed that the agitation at 300 rpm was very favorable to the production, while high frequencies of 600 rpm and 900 rpm showed negative effects on the production of mycelial biomass and pigments. This indicates that high speed of agitation provides high shear rates, causing mycelial rupture and, consequently, reducing the production of pigments. Therefore, in an ideal submerged fungal cultivation, it is necessary to have a balance between the promotion of high oxygen transfer and the maintenance of low hydrodynamic stress in the culture environment (Ochoa and Gomez 2009).

The pigment produced by *F. solani* BRM054066 was submitted to infrared spectroscopy (data not shown), nuclear magnetic resonance spectroscopy, and liquid chromatography coupled to mass spectrometry. Two naphthoquinones with known structures, fusarubin (major compound) and dihydrofusarubin, and an aza-anthraquinone, bostrycoidin, was identified. These compounds belong to the class of quinones, which are highly reactive phenolic compounds capable of interacting with biological systems (Kumagai et al. 2012). The naphtoquinone fusarubin has broad spectrum antimicrobial activity against *Staphylococcus aureus*, *S. epidermis, Escherichia coli, Pseudomonas aeruginosa, Klebsiella pneumoniae, Candida albicans and C. rugosa*. In addition to inhibiting cell viability against six cancer cell lines: MDA-MB-231 (Human breast adenocarcinoma cells, ATCC No. HTB-26), DU145 (Human prostate cancer cells, ATCC No. HTB-81), MCF7 (Human breast adenocarcinoma cells, ATCC No. HTB-22), HeLa (Human cervical cancer cells, ATCC No. CCL-2), B16-F10 (Mouse melanoma cells, ATCC No. CRL-6475) and A549 (Human alveolar adenocarcinoma epithelial cells, ATCC No. CCL-185) (Kumar et al. 2017). In the present work, the pigment of *F. solani* showed potential antioxidant and anti-inflammatory activity, in vitro, at lower concentrations.

The antioxidant activities of natural compounds can be determined using numerous methods, with DPPH free radicals being one of the most commonly used to evaluate this capacity in vitro (Shalaby and Shanab 2013). Khan et al. (2018) evaluated the free radical scavenging activity of compounds isolated from *F. solani* using this DPPH scavenging method. The results showed that the bostrycoidin exhibited significant antioxidant activity with IC_50_ value of 1.6 μg/mL, compared to the IC_50_ values of positive control 1.2, 1.3 and 1.5 for BHA (Butylated hydroxyanisole), trolox and ascorbic acid, respectively. Anhydrofusarubin and fusarubin exhibited antioxidant activity with IC_50_ values of 12.4 and 34.8 μg/mL, respectively. These values may be comparable with the results found in the present study (IC_50_ of 24 μg/mL), due to the presence of fusarubin in the majority of pigment isolated from *F. solani* BRM054066, and a low concentration of bostricoidin.

The relationship between the structure and the biological functions of naphthoquinones has been studied, and the hydroxyl (OH) functional group present in the molecules has been shown to play a key role in eliminating free radicals (Ordoudi et al. 2011; Kumar et al. 2013). The anthraquinone and the naphthoquinones identified in the pigment of *F. solani* BRM054066, in the present study, present two and three hydroxyls, respectively, in their compositions. Thus, the presence of hydroxyls in the quinones identified in the pigment may be responsible for their ability to eliminate free radicals. A study done by Rathna et al. (2016) showed that the crude ethyl acetate extract of the *F. solani* PSC-R too exhibited the ability to eliminate 88% of the DPPH radical at a concentration of 100 μl/mL. Analyses of liquid and gas chromatography coupled to the mass revealed that naphthoquinones, including fusarubin, and phenolic compounds were the main constituents responsible for this activity.

The pigment of *F. solani* BRM054066 also showed anti-inflammatory activity significantly reduce the overexpression of the pro-inflammatory cytokines TNF-α, IL-1β and IL-6 and induce IL-10 and IL-17 anti-inflammatory production by LPS-activated macrophages. Kumar et al. (2017) isolated and identified three naphthoquinones (fusarubin, 3-O-methyl-fusarubin and javanicin) extracted with ethyl acetate from the fungus *Fusarium sp*. These compounds, at concentrations of 5, 10, and 20 μM, were screened for anti-inflammatory activity in THP1 monocytes activated by PMA (phorbol-12-myristate-13-acetate), measuring levels of TNF-α, IL-8 and MCP-1 (monocyte chemoattractant protein-1). It was found that none of these naphthoquinones presented anti-inflammatory activity, by this method. Despite this, dozens of other naphthoquinones, extracted mainly from plants, have been investigated for anti-inflammatory potential. Many of them have presented this property by inhibiting the production of pro-inflammatory mediators, especially nitric oxide, in LPS-activated RAW264.7 macrophages (Yoshida et al. 2010; Pinho et al. 2011; Checker et al. 2014; Dong et al. 2017). The reduction of inflammation gene expression such as inducible NO synthase (iNOS), cyclooxygenase2 (COX-2), interferon-β (IFN-β) can be attributed to the inhibition of multiple targets, such as protein kinases involved in signaling inflammation, like a MAPK p38, and inhibition the transcriptional activator-1 (STAT1) (Yang et al. 2014; Lee et al. 2016).

The pigments obtained from natural sources have the potential to be applied in several industrial sectors (Yusuf et al. 2017). The use of the pigment of *F. solani* BRM054066 as a food additive seems quite promising, since in addition to improving the sensorial characteristics of food and beverages, its components can bring benefits to human health. The combination of its antioxidant and anti-inflammatory effects gives it a protective property of cellular constituents against oxidative stress and against tissue damage caused by inflammatory disorders, thus being able to be effective in the prevention of many diseases. Moreover, the application of this pigment in cosmetics and in the development of drugs also seems quite promising, due to its antioxidant and anti-inflammatory properties identified in the present study, and antibiotic and anticancer action of its components, identified in previous reports (Chowdhury et al. 2017; Kumar et al. 2017). However, further studies are needed to evaluate the physiological and pharmacological effects of this pigment in the human body. A number of factors should be considered, including their safety and toxicity, bioavailability, absorption and distribution, structural transformations and elimination (Yordi et al. 2012).

Due to the increased awareness of the adverse effects associated with synthetic compounds, the search for new technologies to obtain efficient natural compounds is constantly developing. According to the results obtained in the present work, the fungus *F. solani* BRM054066, under optimized conditions of cultivation, proved to be a promising source of biologically active natural pigments with wide industrial applicability.

## Supplementary information

**Additional file 1: Table S1.** Experimental range and factor levels for agitation and cultivation time. **Table S2.** Experimental range and factor levels for glucose concentration and fermentation time. **Table S3.** Comparison of ^1^H and ^13^C NMR data pigment of *F. solani* BRM054066 with data from the literature for fusarubin, bostrycoidin and dihydrofusarubin. **Figure S1.** UPLC-ESI-QTOF chromatogram of pigment extract (ESI- negative ionization mode). High-resolution mass spectrum of the peak at 6.70 min. **Figure S2.** UPLC-ESI-QTOF chromatogram of pigment extract (ESI+ positive ionization mode). High-resolution mass spectrum of the peak at 8.55 min. **Figure S3.**^1^H NMR of pigment extract (600 MHz, CDCl_3_). **Figure S4.**^1^H NMR spectrum of pigment extract (150 MHz, CDCl_3_). **Figure S5.**^1^H,^13^C HSQC NMR spectrum of pigment extract (CDCl_3_, 150 x 600 MHz). **Figure S6.**^1^H,^13^C HMBC NMR spectrum of pigment extract (CDCl_3_, 150 x 600 MHz). **Figure S7.**^1^H,^13^C HSQC and HMBC-based assignment and long-range correlations (*J*^2,3^) observed for fusarubin (A) and dihydrofusarubin (B). **Fig. S8.** Capability of the pigment produced by *F. solani* BRM0540664 to sequester the radical DPPH.

## Data Availability

Not applicable. Not applicable.
