# Supplementary material for: Pigment production by Fusarium solani BRM054066 and determination of antioxidant and anti-inflammatory properties
Source: AMB Express. 2020 Jul 1;10:117. doi: 10.1186/s13568-020-01054-y (PMC7329961; doi:10.1186/s13568-020-01054-y)
Supplement: Supplementary file 1 — Additional file 1: Table S1. Experimental range and factor levels for agitation and cultivation time. Table S2. Experimental range and factor levels for glucose concentration and fermentation time. Table S3. Comparison of 1H and 13C NMR data pigment of F. solani BRM054066 with data from the literature for fusarubin, bostrycoidin and dihydrofusarubin. Figure S1. UPLC-ESI-QTOF chromatogram of pigment extract (ESI- negative ionization mode). High-resolution mass spectrum of the peak at 6.70 min. Figure S2. UPLC-ESI-QTOF chromatogram of pigment extract (ESI+ positive ionization mode). High-resolution mass spectrum of the peak at 8.55 min. Figure S3.1H NMR of pigment extract (600 MHz, CDCl3). Figure S4.1H NMR spectrum of pigment extract (150 MHz, CDCl3). Figure S5.1H,13C HSQC NMR spectrum of pigment extract (CDCl3, 150 x 600 MHz). Figure S6.1H,13C HMBC NMR spectrum of pigment extract (CDCl3, 150 x 600 MHz). Figure S7.1H,13C HSQC and HMBC-based assignment and long-range correlations (J2,3) observed for fusarubin (A) and dihydrofusarubin (B). Fig. S8. Capability of the pigment produced by F. solani BRM0540664 to sequester the radical DPPH. [file 13568_2020_1054_MOESM1_ESM.docx]

**Applied Microbiology and Biotechnology**

**SUPPLEMENTARY MATERIAL**

**Pigment production by *Fusarium solani* BRM054066 and determination of antioxidant and anti-inflammatory properties.**

Bruna S. Menezes^1^; Lucas S. Solidade^1^; Aparecido A. Conceição^1^; Manoel N. Santos Junior^1^; Patrícia L. Leal^1^; Edy S. de Brito^2^; Kirley M. Canuto^2^; Simone Mendonça^3^; Félix G. de Siqueira^3*^; Lucas M. Marques^1*^.

1. Instituto Multidisciplinar em Saúde, Universidade Federal da Bahia. Vitória da Conquista, Bahia, Brasil.

2. Embrapa Agroindústria Tropical, Fortaleza, Ceará, Brasil.

3. Embrapa Agroenergia, Brasília, Distrito Federal, Brasil.

*Corresponding Authors*

**E-mail: lmirandamarques@gmail.com. Phone: +55-77-988265969.*

**E-mail: felix.siqueira@embrapa.br. Phone: +55-61-3448-2324.*

Bruna S. Menezes:

0000-0002-1280-8774

Felix G. De Siqueira:

0000-0001-5239-4994

Lucas M. Marques:

0000-0002-8276-8149

**Table S1.** Experimental range and factor levels for agitation and cultivation time.

| **Factors** | **Range and levels** | | | | | |
| --- | --- | --- | --- | --- | --- | --- |
| **Coded variable, *x_A_*** |  | -0.866 | 0 | 0.866 |  |  |
| **Agitation (rpm), *X_A_*** |  | 0 | 100 | 200 |  |  |
| **Coded variable, *x_T_*** |  | -1 | -0.5 | 0 | 0.5 | 1 |
| **Time (days), *X_T_*** |  | 1 | 4 | 7 | 10 | 13 |

**Table S2.** Experimental range and factor levels for glucose concentration and fermentation time.

| **Factors** | **Range and levels** | | | | | |
| --- | --- | --- | --- | --- | --- | --- |
| **Coded variable, *x_G_*** |  | -1 | -0.5 | 0 | 0.5 | 1 |
| **Glucose (g/L), *X_G_*** |  | 2 | 7 | 12 | 17 | 22 |
| **Coded variable, *x_T_*** |  | -0.866 | 0 | 0.866 |  |  |
| **Time (days), *X_T_*** |  | 3 | 6 | 9 |  |  |

**Table S3.** Comparison of ^1^H and ^13^C NMR data pigment of *F. solani* BRM054066 with data from the literature for fusarubin, bostrycoidin and dihydrofusarubin.

| Fusarubin | | | | Bostrycoidin | | | | Dihydrofusarubin | | | |
| --- | --- | --- | --- | --- | --- | --- | --- | --- | --- | --- | --- |
| ^13^C NMR | | **^1^H NMR** | | **^13^C NMR** | | **^1^H NMR** | | **^13^C NMR** | | **^1^H NMR** | |
| δ Exp. | **δRef. ^#^** | **δ Exp.** | **δRef. *** | **δC Exp.** | **δC Ref. ^+^** | **δH Exp.** | **δH Ref ^+^** | **δC Exp.** | **δC Ref.*** | **δH Exp.** | **δH Ref ^&^** |
| 184.9 | -^a^ | 12.93 (OH) | 12.91 (OH) | -^b^ | 186.4 | 13.49 (1H, s) | 13.49 (1H, s) | 206.2 | 202.8 | 12.20 (OH) | 12.22 (OH) |
| 178.5 | -^a^ | 12.65 (OH) | 12.65 (OH) | -^b^ | 183.9 | 13.20(1H, s) | 13.20(1H, s) | 203.8 | 198.8 | 12.01 (OH) | 12.05 (OH) |
| 160.9 | 160.6 | 6.18 (1H, s) | 6.17 (1H, s) | -^b^ | 165.4 | 6.76 (1H, s) | 6.76 (1H, s) | 157.8 | 157.6 | 6.67 | 6.68 |
| 160.5 | 160.6 | 4.89 (2H, s) | 5.01; 4.76 (1H, dm) | -^b^ | 161.3 | 9.50 (1H, s) | 9.50 (1H, s) | 156.9 | 156.7 | 4.24; 4.17 | 4.22; 4.22 |
| 157.9 | 156.7 | 3.94 (3H, s) | 3.93 (3H, s) | -^b^ | 157.9 | 7.96 (1H, s) | 7.96 (1H, s) | 146.3 | 146.1 | 3.96 (OCH_3_) | 3.97 (OCH_3_) |
| 137.3 | 137.1 | 3.04; 2.71 (1H, d, 18 Hz) | 3.06; 2.68 (1H, d, 18 Hz) | -^b^ | 151.2 | 4.02 (3H, s) | 4.02 (3H, s) | 114.1 | 114.1 | 2.96 | 2.97 |
| 133.0 | 133.0 | 1.65 (3H, s) | 1.65 (3H, s) | -^b^ | 143.3 | 2.80 (3H, s) | 2.80 (3H, s) | 107.3 | 107.3 | 3.40 | 3.43 |
| 110.9 | 110.0 |  |  | -^b^ | 138.7 |  |  | 106.7 | 106.3 | 2.44; 1.68 | 2.42; 1.69 |
| 109.9 | 109.5 |  |  | -^b^ | 124.6 |  |  | 95.5 | 97.4 | 1.54 | 1.54 |
| 107.8 | 107.4 |  |  | -^b^ | 118.0 |  |  | 59.5 | 59.5 |  |  |
| 94.5 | -^a^ |  |  | -^b^ | 107.9 |  |  | 56.9 | 56.5 |  |  |
| 58.8 | 58.2 |  |  | 56.8 | 56.8 |  |  | 46.2 | 45.9 |  |  |
| 56.9 | 56.6 |  |  | 25.3 | 25.3 |  |  | 43.6 | 43.3 |  |  |
| 32.4 | 32.4 |  |  |  |  |  |  | 34.4 | 35.1 |  |  |
| 29.6 | 22.5 |  |  |  |  |  |  | 30.1 | -^a^ |  |  |

**δ Exp** = Experimental values; **δ Ref** = Reference values from the literature.^a^ Unreported data.

Kumar et al. (2017) (**#**) and Kurobane et al., 1985 (*****). Values reported by Yamamoto et al., 2002 (^+^) and Tatum & Baker, 1983 (^&^)

**Figure S1**- UPLC-ESI-QTOF chromatogram of pigment extract (ESI- negative ionization mode). High-resolution mass spectrum of the peak at 6.70 min.

**Figure S2**- UPLC-ESI-QTOF chromatogram of pigment extract (ESI+ positive ionization mode). High-resolution mass spectrum of the peak at 8.55 min.

**Figure S3**- ^1^H NMR of pigment extract (600 MHz, CDCl_3_)

**Figure S4**- ^1^H NMR spectrum of pigment extract (150 MHz, CDCl_3_)


 **Figure S5**- ^1^H,^13^C HSQC NMR spectrum of pigment extract (CDCl_3_, 150 x 600 MHz)

**Figure S6**- ^1^H,^13^C HMBC NMR spectrum of pigment extract (CDCl_3_, 150 x 600 MHz)

**(A)**

**(B)**

**Figure S7.** ^1^H,^13^C HSQC and HMBC-based assignment and long-range correlations (*J*^2,3^) observed for fusarubin (A) and dihydrofusarubin (B).

**
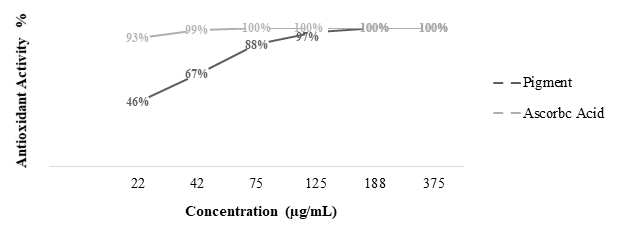
**

**Figure S8.** Capability of the pigment produced by *F. solani* BRM0540664 to sequester the radical DPPH.

**SUPPLEMENTARY MATERIAL REFERENCES**

Kumar KP, Javvaji K, Poornachandra Y, Devi Allanki AD, Misra S (2017) Antimicrobial, Anti-plasmodial and Cytotoxicity Properties of Bioactive Compounds from Fusarium sp. USNPF102. J Microbiol Res. 7(2):23-30. doi:10.5923/j.microbiology.20170702.01

Kurobane I, Zaita N, Fukuda A (1985) New metabolites of Fusarium Marti related to dihydrofusarium. J. Antibiotics. 29:205-214. doi:10.7164/antibiotics.39.205

Tatum JH, Baker RA (1983) Naphtoquinones produced by Fusarium solani isolated from Citrus. Phyochemistry. 22:543-547. doi:10.1016/0031-9422(83)83042-8

Yamamoto Y, Kinoshita Y, Ran Thor G, Hasumi M, Kinoshita K, Koyama K, Takahashi K, Yoshimura I (2002) Isofuranonaphthoquinone derivatives from cultures of the lichen Arthonia cinnabarin a(DC.) Wallr. Phytochemistry. 60:741-745. doi:10.1016/s0031-9422(02)00128-0
